# Low Glucose Mediated Fluconazole Tolerance in *Cryptococcus neoformans*

**DOI:** 10.3390/jof7060489

**Published:** 2021-06-18

**Authors:** Somanon Bhattacharya, Natalia Kronbauer Oliveira, Anne G. Savitt, Vanessa K. A. Silva, Rachel B. Krausert, Berhane Ghebrehiwet, Bettina C. Fries

**Affiliations:** 1 Division of Infectious Diseases, Department of Medicine, Stony Brook University, Stony Brook, NY 11794, USA; Somanon.Bhattacharya@stonybrookmedicine.edu (S.B.); VanessaKarina.AlvesDaSilva@stonybrookmedicine.edu (V.K.A.S.); 2 Department of Microbiology and Immunology, Renaissance School of Medicine, Stony Brook University, Stony Brook, NY 11794, USA; natalia.kronbauerdeoliveir@stonybrook.edu (N.K.O.); anne.savitt@stonybrook.edu (A.G.S.); Rachel.krausert@stonybrook.edu (R.B.K.); 3 Division of Rheumatology, Allergy and Immunology, Department of Medicine, Stony Brook University, Stony Brook, NY 11794, USA; Berhane.Ghebrehiwet@stonybrookmedicine.edu; 4 Veterans Administration Medical Center, Northport, NY 11768, USA

**Keywords:** azole resistance, efflux pumps, fungal pathogenesis, cryptococcosis, low glucose, glucose starvation, mitochondria

## Abstract

Chronic meningoencephalitis is caused by *Cryptococcus neoformans* and is treated in many parts of the world with fluconazole (FLC) monotherapy, which is associated with treatment failure and poor outcome. In the host, *C. neoformans* propagates predominantly under low glucose growth conditions. We investigated whether low glucose, mimicked by growing in synthetic media (SM) with 0.05% glucose (SM^lowglu^), affects FLC-resistance. A > 4-fold increase in FLC tolerance was observed in seven *C. neoformans* strains when minimum inhibitory concentration (MIC) was determined in SM^lowglu^ compared to MIC in SM with normal (2%) glucose (SM^nlglu^). In SM^lowglu^, *C. neoformans* cells exhibited upregulation of efflux pump genes *AFR1* (8.7-fold) and *AFR2* (2.5-fold), as well as decreased accumulation (2.6-fold) of Nile Red, an efflux pump substrate. Elevated intracellular ATP levels (3.2-fold and 3.4-fold), as well as decreased mitochondrial reactive oxygen species levels (12.8-fold and 17-fold), were found in the presence and absence of FLC, indicating that low glucose altered mitochondrial function. Fluorescence microscopy revealed that mitochondria of *C. neoformans* grown in SM^lowglu^ were fragmented, whereas normal glucose promoted a reticular network of mitochondria. Although mitochondrial membrane potential (MMP) was not markedly affected in SM^lowglu^, it significantly decreased in the presence of FLC (12.5-fold) in SM^nlglu^, but remained stable in SM^lowglu^-growing *C. neoformans* cells. Our data demonstrate that increased FLC tolerance in low glucose-growing *C. neoformans* is the result of increased efflux pump activities and altered mitochondrial function, which is more preserved in SM^lowglu^. This mechanism of resistance is different from FLC heteroresistance, which is associated with aneuploidy of chromosome 1 (Chr1).

## 1. Introduction

Cryptococcal meningitis (CM) is caused predominantly by *Cryptococcus neoformans* and less frequently by *Cryptococcus gattii* [1,2]. A global data analysis estimates that this life-threatening fungal infection causes 180,000 deaths annually. Although CM is primarily diagnosed in patients with advanced HIV/AIDS, rising numbers of CM cases have also been reported in immune-competent patients [3,4]. Amphotericin B with or without 5-fluorocytosine (5FC) for two weeks followed by fluconazole (FLC) maintenance therapy [5] is the recommended treatment. However, FLC monotherapy remains the only option in many countries with limited resources. FLC monotherapy is associated with higher relapse rates. Even with antifungal therapy, CM mortality can exceed 50% [6,7,8].

FLC is a fungistatic triazole that inhibits lanosterol 14α-demethylase encoded by *ERG11* in the ergosterol biosynthesis pathway [9]. The relationship between FLC resistance and CM treatment failure is only partially understood. FLC resistance is measured by minimum inhibitory concentration (MIC) assay. Baseline FLC resistance for *C. neoformans* is defined as FLC MIC > 8, which is rarely observed in clinical *C. neoformans* strains [10]. FLC resistance in *C. neoformans* is mediated by augmentation of efflux pump activity and/or changes in the ergosterol target (encoded by *ERG11*), similar to FLC resistance in *Candida* sp. [11,12,13,14,15]. The efflux pumps that are associated with FLC resistance in *C. neoformans* are Afr1, Afr2, and Mdr1 [13,16], which all require adenosine tri-phosphate (ATP) to pump FLC out of the cell. The majority of ATP synthesis occurs in mitochondria, and loss or disruption of mitochondrial function has been linked to FLC resistance in several pathogenic fungi [17,18]. Treatment failure of FLC monotherapy regimens is linked to FLC heteroresistance, which is an intrinsic property of all *C. neoformans* strains. Heteroresistance is exhibited in a subpopulation and the emerging resistance under FLC selection pressure is therefore defined as FLC tolerance. Heteroresistance is associated with aneuploidy of chromosome 1 (Chr1), which harbors the genes encoding *ERG11* and *AFR1* [19,20].

During chronic infection of humans, *C. neoformans* most commonly resides in the lungs, brain, and cerebrospinal fluid (CSF), all of which constitute low glucose growth environments [21] to which *C. neoformans* must adapt. Although *C. neoformans* can rely on alternative carbon sources [22], major metabolic adaptation has to occur. Interestingly, altered FLC tolerance has been described in mutants of key regulators in the TOR pathway, which is activated in glucose starvation [23]. Indeed, transcriptome analysis of fungal isolates from HIV/AIDS patients has correlated with elevated expression of *STL1,* a carbon nutrient transporter causing failure to clear the fungus rapidly from CSF. Similarities in gene expression profiles of *C. neoformans* grown in nutrient-deprived medium and *C. neoformans* cells grown in pooled CSF have also been described previously [24,25]. Interestingly, low glucose also promotes longevity of *C. neoformans* populations [1] similar to *Saccharomyces cerevisiae* [26]. Earlier studies from our laboratory with *C. neoformans* cells of advanced generational age showed that these old cells exhibit increased tolerance to FLC [27] compared to their respective young daughter cells. Since generationally aged *C. neoformans* cells accumulate in the host environment predominantly under low glucose growth conditions and are FLC tolerant, it became important to evaluate the effects of glucose starvation on FLC tolerance independent of aging.

In the present study, we therefore sought to specifically explore the role of a low glucose growth environment on FLC tolerance. We compared the tolerance to FLC and efflux efficacy of wild type and respective mutants under low and standard glucose growth conditions. We analyzed mitochondrial function and structure under these conditions as they are critical for ATP production, which fuels these efflux pumps. Our data indicate that even in the absence of replicative aging, low glucose itself causes upregulation of efflux pumps. Enhanced efflux pump function is not associated in these conditions with the change of gene copy numbers that encode the efflux pump genes. Instead, low glucose growth conditions lead to improved mitochondrial function and ATP production, all of which contribute to markedly enhanced efflux pump function leading to FLC resistance.

## 2. Materials and Methods

### 2.1. Media and Growth Conditions

All the strains listed in Table S1 were first streaked in yeast peptone dextrose (YPD, Becton Dickinson, Franklin Lakes, NJ, USA) agar plate and incubated at 37 °C for 48 h. A single colony of each strain was suspended in synthetic medium (SM) containing 1.7 g yeast nitrogen base without amino acids (BD),1 g Drop-Out mix (USBiological LifeSciences, Salem, MA, USA), 0.4% ethanol, 5 g (NH_4_)_2_SO_4_, 3.3 g NaCl, with normal (2%) glucose (SM^nlglu^ ) or low (0.05%) glucose (SM^lowglu^), and was incubated at 37 °C overnight. The next day the cells were counted, and exponentially growing cells were used for the following assays. For assays in the presence of FLC, the overnight cell cultures were diluted and grown exponentially in the presence of 1 μg/mL FLC, and then used for all the assays. 30% glycerol stocks of all listed strains were stored at −80 °C for future use. Except for the minimum inhibitory assays, all the assays were performed using cells from the KN99α strain.

### 2.2. Analysis of Drug Resistance Gene and Super-Oxide dismutase (SOD) Expression

RNA was isolated from exponentially growing cells in both SM^nlglu^ and SM^lowglu^ using RNEASY Plus Kit (Qiagen, Germantown, MD, USA) following the manufacturer’s protocol. RNA was quantified using BioSpectrophotometer (Eppendorf, Enfield, CT, USA). An absorbance ratio (A260/A280) of 2.0 or greater was considered pure and good quality RNA. Next, 250 ng of RNA was converted to cDNA using Verso cDNA kit (Thermo Fisher Scientific, Waltham, MA, USA) following the manufacturer’s guidelines. Oligo-dT was used as a primer for generating cDNA. Prepared cDNA was then diluted 1:5 with RNAse/DNAse free water (HyClone Laboratories, Logan, UT, USA) and used for gene expression analysis by qPCR (Roche Life Science, Indianapolis, IN, USA) using Power Sybr Green Master Mix (Applied Biosystems, Foster City, CA, USA) following the manufacturer’s protocols. Oligonucleotides used to analyze the gene expression of the drug resistance genes, *AFR1*, *AFR2*, *MDR1*, *ERG11*, and SODs, cytoplasmic SOD (*CNAG_04388*) and mitochondrial SOD (*CNAG_01019*) are listed in Table S2. House-keeping gene *ACT1*, which encodes β-actin, was used as an internal control. Data were normalized to the gene expression in SM^nlglu^ medium and were calculated with a 2^−ΔΔCt^ method as previously published [28]. qPCR was performed in biological triplicate and fold changes greater than 2.0 and *p* < 0.05 were considered significant. Melting curves were performed after the qPCR reactions to determine the specificities of the oligonucleotides used.

Previously published transcriptome from our laboratory was reanalyzed for this study [29].

### 2.3. Nile Red Efflux Assay

A Nile Red assay was performed to analyze the efflux activities of the membrane transporters of *Cryptococcus neoformans* in SM^nlglu^ and SM^lowglu^ media. Briefly, the cells were exponentially grown in the respective media. After growth, the cells were counted, and 10^7^ cells were used for the Nile Red assay. First, the cells were washed three times with 1X PBS to remove any residual glucose. The cells were then starved for 2 h in PBS. After starvation, Nile Red was added to the cells at a final concentration of 7 µM. The efflux was initiated after the addition of 2% or 0.05% glucose to the starved cells. Accumulation of Nile Red was measured using fluorescence plate reader.

Fluorescence was measured at 0 min and 30 min using λ_ex_ of 553 nm and λ_em_ of 636 nm. Accumulation was calculated by monitoring the decrease in the fluorescence in the supernatant over 30 min period. More accumulation of the dye at the end of 30 min period signifies lesser efflux.

### 2.4. Measurement of Mitochondrial Reactive Oxygen Species (mROS), Mitochondrial Membrane Potential (MMP) and Mitochondrial Mass

For measuring mROS, exponentially growing cells under both SM^nlglu^ and SM^lowglu^ conditions in the presence or absence of FLC were first washed three times with 1X PBS. After washing, the cells were counted and 10^6^ cells for all the conditions were stained with MitoSOX Red (Thermo Scientific, Waltham, MA, USA) following the manufacturer’s guidelines. Briefly, on the day of the experiment, a fresh vial of MitoSOX Red was resuspended with 13 µL dimethyl sulfoxide (DMSO) to make the stock solution concentration at 5 mM. Then, 10^6^ cells were stained with MitoSOX Red at a final concentration of 500 nM and incubated for 15 min at 37 °C in dark. After staining, the cells were washed three times with 1X PBS and 200 µL of washed stained cells were loaded in black 96 well plates (Costar, Corning Life Sciences, Tewksbury, MA, USA). Fluorescence was read in a fluorescent plate reader (SpectraMax I3X, Molecular Devices LLC, San Jose, CA, USA) with λ_ex_ of 510 nm and λ_em_ of 580 nm. More fluorescence signifies more mROS levels under SM^nlglu^ or SM^lowglu^ conditions in the presence or absence of FLC. Unstained cells were used as a negative control. Fluorescence was measured in relative fluorescence units (RFU) by subtracting the RFU values between the stained and non-stained cells (negative control). The assay was performed in biological triplicate.

For measuring MMP, exponentially growing cells from both SM^nlglu^ and SM^lowglu^ conditions in the presence or absence of FLC were first washed three times with PBS. After washing, the cells were counted, and 10^6^ cells were stained with MitoTracker Deep Red (Thermo Scientific, Waltham, MA, USA) following the manufacturer’s guidelines. Briefly, on the day of the experiment, one vial of MitoTracker Deep Red was resuspended in DMSO to a concentration of 1 mM. Then, 10^6^ cells (SM^nlglu^ and SM^lowglu^) with or without FLC were stained with MitoTracker Deep Red at a final concentration of 0.2 µM for 30 min at 37 °C in dark. The stained cells were then washed three times with PBS and 200 µL of washed stained cells were loaded in black 96 well plates (Costar). Fluorescence was read in a fluorescent plate reader (SpectraMax I3X, Molecular Devices LLC, San Jose, CA, USA) with an λ_ex_ of 644 nm and an λ_em_ of 665 nm. More fluorescence signifies more MMP under the tested conditions. Unstained cells were used as a negative control. Fluorescence was measured in RFU by subtracting the RFU values between the stained and non-stained cells (negative control). The assay was performed in biological triplicate.

For measuring the mitochondrial mass, exponentially growing cells from both SM^nlglu^ and SM^lowglu^ conditions +/− FLC were washed 3× with PBS. After washing, 10^6^ cells from all conditions were stained with MitoTracker Green FM (Thermo Scientific, Waltham, MA, USA) following the manufacturer’s protocol. Briefly, on the day of the experiment, a fresh vial of MitoTracker Green was resuspended in DMSO to make a 1 mM stock solution. MitoTracker Green was then added to the 10^6^ cells from SM^nlglu^ and SM^lowglu^ media +/− FLC at a final concentration of 200 nM and incubated at 37 °C for 30 min. Fungal cells were washed 3× with PBS, and 200 µL of stained cells were loaded in a black 96 well plate (Costar, Corning Life Sciences, Tewskbury, MA, USA). Fluorescence was read in a fluorescent plate reader (SpectraMax I3X, Molecular Devices LLC, San Jose, CA, USA) with an λ_ex_ of 490 nm and an λ_em_ of 516 nm. The assay was performed in biological triplicate.

### 2.5. Fluorescence Microscopy and Transmission Electron Microscopy

Fungal cells grown under the various conditions and stained with MitoTracker Green FM were placed on coverslips and fixed with ice-cold 100% methanol for 10 min at −20 °C. Methanol was removed and the coverslips allowed to air dry without washing. Coverslips were then mounted on slides using Vectashield plus DAPI (4′,6-diaminidino-2-phenylindole) mounting medium (Vector Labs, Burlingame, CA, USA) and sealed. Stained fungal cells were visualized on an inverted/DIC Zeiss Axiovert 200M microscope equipped with an AxioCam HRm camera (Zeiss, Thornwood, NY, USA) and mercury arc lamp light source using a 100× Plan-Apochromat (numerical-aperture 1.40 oil objective) at an optovar setting of 1.6 and operated using Axiovision version 4.8 (Zeiss) software. The following excitation and emission wavelengths were used for imaging: for DAPI, λ_ex_ of 360 ± 20 nm, λ_em_ of 460 ± 25 nm; for FITC, λ_ex_ of 480 ± 20 nm, λ_em_ of 535 ± 25 nm; Images were deconvolved using the inverse filter method (AxioVision 4.8). Each experiment was repeated at least once on different days keeping all the conditions the same.

For transmission electronic microscopy (TEM) analysis, 10^7^cells from both SM^nlglu^ and SM^lowglu^ media were fixed in 3% glutaraldehyde in 0.1M sodium cacodylate buffer pH 7.4 at 4 °C overnight. After, yeast cells were washed 3× with 0.1 M phosphate buffer and were dispersed in ultra-low temperature agarose at 4 °C for 30 min. Subsequently, the agarose samples were cut into small cubes, and the pieces were post-fixed in aqueous 1% KMnO_4_ for 1h at room temperature (RT). Blocks were rinsed with dH_2_O and treated with 0.5% sodium meta-periodate for 15 min at RT to allow infiltration. Then, blocks were rinsed again with dH_2_O and underwent dehydration steps through a graded ethanol series (30%, 50%, 70%, 95%, 100%). Samples were treated with 100% propylene oxide, embedded with Spurr’s Resin, and polymerized in an oven at 60 °C for 24 h. Ultrathin sections (80 nm) were cut using an ultramicrotome (Leica EM UC7, Leica Microsystems, Buffalo Grove, IL, USA), placed on 300 mesh grids, and contrasted with uranyl acetate and lead citrate. Images were acquired at magnifications 18,500× and 49,000× using a FEI TeCnai 12 BioTwinG^2^ TEM and AMT XR 60 CCD digital camara system [30]. TEM was performed at the Central Microscopy Imaging Center at Stony Brook University. Images were analyzed in Fiji [31]. 40 mitochondria from the cells grown under each condition were measured from 30,000× zoom TEM images with a 500 nm scale bar.

### 2.6. Measurement of Cellular ATP Levels

Cellular ATP levels were quantified using ATP Bioluminescent Assay Kit (Sigma-Aldrich, St. Louis, MO, USA) following the manufacturer’s guidelines. Briefly, exponentially growing cells under both SM^nlglu^ and SM^lowglu^ conditions in the presence or absence of FLC were washed three times with PBS. The cellular ATP was first isolated with the trichloroacetic acid method (TCA) as previously published [32]. 10^7^ cells were mixed with 5% TCA and alkaline lysis solution (1% sodium dodecyl sulfate and 0.2 N NaOH) and vortexed for 15 min in the presence of sterile acid-washed glass beads to initiate lysis. After vortexing, the cell lysates without the glass beads were transferred and boiled at 100 °C for 10 min, cooled and diluted 1:50 with ATP assay mix dilution buffer supplied with the ATP Bioluminescent Assay Kit (Sigma-Aldrich, St. Louis, MO, USA). Diluted sample was mixed with 100 µL of ATP assay mix solution supplied with the ATP Bioluminescent Assay Kit in white-opaque bioluminescent 96 well plates (Corning-Costar, Cambridge, MA, USA). The mixture was incubated in the dark for 3 min and then bioluminescence was measured with the spectrophotometer (SpectraMax I3X). ATP standard solution was used in serial dilution to generate a standard curve. This standard curve was used to interpolate the cellular ATP levels. The assay was performed in biological replicate.

### 2.7. Minimum Inhibitory Concentration

Minimum inhibitory concentrations (MIC) were performed using modified CLSI recommendations [33] with C. neoformans cells grown exponentially in SM medium (supplemented with 2%, 0.5%, and 0.05% glucose). After growth, the cells were counted, and 10^5^ cells per well were used as inoculum to analyze the MICs. The MICs were performed using flat-bottomed 96 well plates (Corning-Costar). In the 96 well plates, FLC was serially diluted two-fold in all the tested media starting at 128 µg/mL in column 1. FLC was not added in Column 11, which served as a growth control (GC) for the MIC assays. 10^5^ cells were added in columns 1 through 11. The plates were incubated at 37 °C for 72 h. After incubation, optical density (OD) at 600 nm was measured and MICs were calculated by comparing the ODs of wells containing FLC with the OD of GC. MICs were performed in biological triplicate. MIC_80_ was determined, which is defined as the lowest concentration of antifungal drugs that can inhibit 80% of fungal cell growth.

### 2.8. Analysis of Gene Copy Number

Gene copy number was analyzed with qPCR following a previously published protocol [12]. First, genomic DNA was isolated from exponentially growing cells in SM^nlglu^ and SM^lowglu^ media. qPCR was then used on the genomic DNA to analyze the gene copy number of *AFR1* and *AFR2* genes using the oligonucleotides listed in Table S2. *ACT1* gene was used as an internal control. The assay was done in triplicate.

### 2.9. Statistics

All statistical analysis was performed using Prism 9.0 (GraphPad, San Diego, CA, USA). The individual statistical tests performed in each assay are described in the figure legends.

## 3. Results

### 3.1. Low Glucose Increases FLC Resistance and Efflux Pump Activity

**Figure 1:**
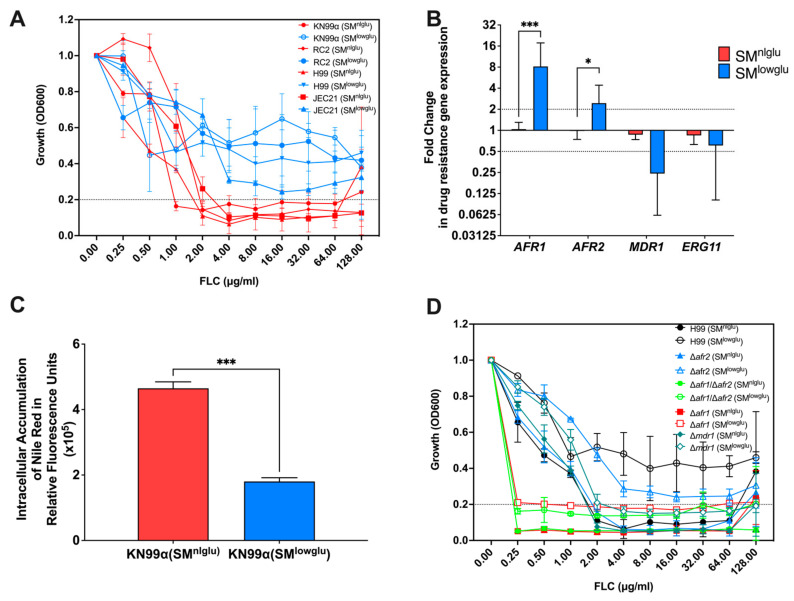
Effects of low glucose in FLC resistance. (**A**) In the presence of low glucose, *C. neoformans* cells are more resistant to FLC; blue lines signify MICs of different *C. neoformans* strains in low glucose media, SM^lowglu^; red lines signify MICs of different *C. neoformans* strains in normal glucose media, SM^nlglu^. Error bars represent the standard deviation between biological triplicates. Dotted line signifies MIC80 cut offs for the respective strains. (**B**) Expression of ABC transporters encoding genes, *AFR1*, *AFR2*, and *MDR1*, and FLC drug target encoding gene *ERG11* in SM^nlglu^ (red bar) and SM^lowglu^ (blue bar) media. qPCR was used to analyze the expression of genes in KN99α cells grown in SM^nlglu^ and SM^lowglu^ media. The data were normalized to the gene expression in the cells grown in SM^nlglu^ media. *ACT1* was used as an internal control for the qPCR. The dotted lines signify two-fold up- or downregulation of the respective genes. qPCR was performed in biological triplicate and error bars signify standard deviations in the triplicates. Student’s *t*-test with Welch’s correction was performed to determine the *p*-value (* *p* < 0.05; *** *p* < 0.001). (**C**) Nile Red accumulation assay to determine efflux efficiencies of the ABC transporters in KN99α cells in SM^nlglu^ (red bar) and SM^lowglu^ (blue bar) media. Higher accumulation signifies lower efficiency of efflux. The assay was performed in biological triplicate. Error bars signify the standard deviations between the replicates. Student’s *t*-test with Welch’s correction was performed to determine the *p*-value (*** *p* < 0.001). (**D**) MICs of the mutants encoding the efflux pump genes in SM^nlglu^ (closed symbols) and SM^lowglu^ (open symbols) were analyzed. Error bars signify standard deviations between the biological triplicate. Dotted line signifies MIC80 cut offs for the respective strains.

This study explored FLC resistance at a very low glucose concentration (0.05%) because low glucose growth conditions are encountered in the host in the lung and spinal fluid. Such low glucose growth conditions mildly prolong doubling time (2.8 h versus 2.1 h, respectively) but greatly promote longevity [1,27]. Here, we sought to investigate if low glucose by itself affects FLC tolerance. To test this, we performed minimum inhibitory concentrations (MIC) in a synthetically defined medium supplemented with normal (2%) glucose (SM^nlglu^), 0.5% glucose, and low (0.05%) glucose (SM^lowglu^). MICs were performed on four laboratory *C. neoformans* strains (KN99α, RC2, H99, and JEC21) and four low-passaged clinical isolates (I55, I114, J9 and J22), all of which were sensitive to FLC at baseline level under 2% glucose conditions (FLC MIC < 2 μg/mL, Figure 1A, Table 1). Interestingly, all four laboratory strains showed markedly increased FLC MICs when MICs were assessed in low glucose (0.05% glucose) conditions (FLC MIC > 64 μg/mL, Table 1, Figure 1A) but not when the medium was supplemented with 0.5% or 2% glucose (Table 1). Increased FLC MIC (4-fold) was also observed in three out of four low passaged clinical isolates in SM^lowglu^ medium (Table 1). In the remaining clinical isolate, J9, FLC MIC only increased 2-fold in SM^lowglu^ medium (Table 1).

Given that growth is only mildly impaired, we explored other mechanisms for enhanced FLC tolerance including efflux pump activities and/or alterations in the FLC target enzyme encoded by *ERG11*. First, we analyzed the gene expression of three *C. neoformans* efflux pumps, Afr1, Afr2, and Mdr1, and azole drug target encoding gene *ERG11.* Under low glucose conditions, a significant increase in expression of the efflux pump encoding genes *AFR1* (8.7-fold, *p* = 0.001) and *AFR2* (2.5-fold, *p* = 0.041) was observed (Figure 1B), whereas expression of *MDR1* and *ERG11* was unchanged relative to normal glucose conditions (Figure 1B). Second, a subanalysis of our previously published transcriptome of *C. neoformans* [1] grown in low glucose conditions was done that revealed 10 of 23 genes encoding enzymes of the ergosterol pathway were downregulated under low glucose growth conditions, while the genes encoding the remaining 13 enzymes were unchanged under low glucose (Table S3). Next, efflux pump activities were assessed by measuring the accumulation of Nile Red dye after uptake by *C. neoformans* cells as described previously [13]. These assays confirmed a significant decrease in intracellular accumulation of Nile Red in KN99α cells grown in SM^lowglu^ when compared to cells grown in SM^nlglu^ (1.8 × 10^5^ RFU vs. 4.6 × 10^5^ RFU, *p* = 0.001; Figure 1C).

To identify which amongst the three efflux pumps are important for low glucose-mediated FLC tolerance, we performed FLC MICs on the respective mutants Δ*afr1,* Δ*afr2,* Δ*mdr1* as well as the double mutant Δ*afr1/*Δ*afr2* in SM^nlglu^ and SM^lowglu^ media. Previously, it was reported that Δ*afr1* and the double mutant Δ*afr1/*Δ*afr2* exhibit MICs of 0.25 μg/mL which is 8-fold lower than that of the parent wild type H99 strain. In contrast, Δ*afr2* exhibits a MIC of 2 μg/mL, which is unchanged from that of H99. In SM^lowglu^ medium, the MICs of Δ*afr1* and the Δ*afr1/*Δ*afr2* exhibited MICs similar to that under SM^nlglu^ medium conditions (0.25 to 0.5 μg/mL; Figure 1D, Table 1). This implies that Afr1 alone or in combination with Afr2 efflux pumps are responsible for increased FLC tolerance in SM^lowglu^ medium. The Δ*afr2* mutant FLC MIC in SM^lowglu^ medium was similar to that of wild type (Table 1, Figure 1D) suggesting that Afr2 did not contribute to emerging FLC resistance in low glucose growth conditions. Interestingly, Δ*mdr1* mutant exhibited wild-type MIC to FLC that was also not affected by lowering glucose (Table 1, Figure 1D).

Lastly, we examined whether the enhanced expression of *AFR1* and *AFR2* was driven by changes in gene copy number [20]. *AFR1* and *MDR1* are located on Chromosome1 and *AFR2* is located on Chromosome5. Based on Chromosome 1and Chromosome 5-genomic sequences, we designed probes that only amplified genomic gene copies, which therefore also reflect the copy number of these two chromosomes. These data demonstrate no change in copy numbers for *AFR1* and *AFR2* genes in cells in SM^nlglu^ or SM^lowglu^ medium (Figure S1).

### 3.2. Effects of Low Glucose on Mitochondrial Function

**Figure 2:**
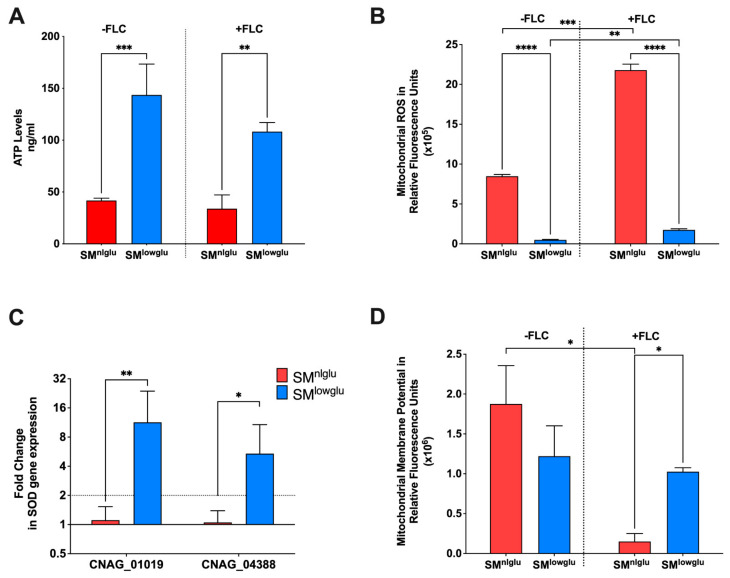
Effects of low glucose on mitochondrial function. (**A**) In SM^lowglu^ media, cellular ATP levels were increased in KN99α; blue bar signifies cellular ATP levels in SM^lowglu^ medium; red bar signifies cellular ATP levels in SM^nlglu^ medium. The assay was performed in biological triplicate in the presence (+FLC) or absence (−FLC) of 1 μg/mL FLC. Error bars signify the standard deviation of the replicates. Student’s *t*-test with Welch’s correction was performed to determine the *p*-value (** *p* = 0.0013, *** *p* = 0.0002). (**B**) Under low glucose, mitochondrial ROS (mROS) levels were lower; blue bar signifies mROS levels in SM^lowglu^ medium; red bar signifies mROS levels in SM^nlglu^ medium. The assays were performed in the presence (+FLC) or absence (−FLC) of 1 μg/mL FLC. The assay was performed in biological triplicate. Error bars signify the standard deviation of the triplicates. Student’s *t*-test with Welch’s correction was performed to determine the *p*-value (**** *p* < 0.0001, *** *p* = 0.004, ** *p* = 0.0022). (**C**) Under low glucose, the expression of genes encoding superoxide dismutases (SOD) was significantly higher. the blue bar signifies gene expression under SM^lowglu^ medium; the red bar signifies gene expression under SM^nlglu^ medium. qPCR was used to analyze the expression of SODs in KN99α cells grown in SM^nlglu^ and SM^lowglu^ media. *CNAG_01019* encodes mitochondrial SOD while *CNAG_04388* encodes cytoplasmic SOD. The data were normalized to the gene expression in the cells grown in SM^nlglu^ medium. *ACT1* was used as an internal control for the qPCR. The dotted lines signify two-fold up- or downregulation of the respective genes. qPCR was performed in biological triplicate and error bars signify standard deviations in the replicates. Student’s *t*-test with Welch’s correction was performed to determine the *p*-value (** *p* = 0.0063, * *p* = 0.027). (**D**) Under low glucose, mitochondrial membrane potential (MMP) was lower; blue bar signifies MMP levels in calorie restriction SM^lowglu^ medium; red bar signifies MMP levels in normal SM^nlglu^ medium. The assays were done in the presence (+FLC) or absence (−FLC) of 1 μg/mL of FLC. The assay was performed in biological triplicate. Error bars signify the standard deviation of the triplicate values. Student’s *t*-test with Welch’s correction was performed to determine the *p*-value (* *p* < 0.05).

The proper function of the aforementioned efflux pumps requires energy in form of ATP for transporting drugs and other xenobiotics. Cellular ATP levels were quantified with a commercial kit in cells grown in SM^nlglu^ and SM^lowglu^ media. These experiments showed that ATP levels were 3.4-fold higher in the cells grown under SM^lowglu^ medium when compared to the intracellular ATP levels in the cells grown in SM^nlglu^ medium (143.6 ng/mL vs. 41.8 ng/mL; *** *p* = 0.0002, Figure 2A). A similar fold change in ATP levels in cells grown in SM^lowglu^ and SM^nlglu^ media in the presence of FLC was also documented (3.2-fold; 108.3 ng/mL vs. 33.9 ng/mL; ** *p* = 0.0013; Figure 2A). Published transcriptome data from cells grown in low glucose indicated that four genes encoding fatty acid oxidation associated enzymes (*CNAG_04485, CNAG_00984, CNAG_03666, CNAG_04688*) were significantly upregulated (Table 2) when compared with the cells grown in normal glucose medium. These data suggest that increased fatty acid oxidation in *C. neoformans* cells grown under low glucose may contribute to the observed increase in cellular ATP levels.

Since the majority of ATP is synthesized in mitochondria, additional investigations were undertaken to assess the mitochondrial function of cells grown in SM^nlglu^ and SM^lowglu^ media. First, the mROS levels were analyzed by staining the cells with MitoSOX fluorescent dye and quantified by measuring fluorescence intensity. mROS levels were significantly lower (~16-fold) in KN99α cells grown in SM^lowglu^ medium when compared to mROS levels in KN99α cells grown in SM^nlglu^ medium (0.5 × 10^5^ RFU vs. 8.5 × 10^5^ RFU, **** *p* < 0.0001; Figure 2B). Treatment with sub-therapeutic FLC increased mROS production in both 2–3 fold (21.8 × 10^5^ RFUvs 8.5 × 10^5^ RFU in SM^nlglu^ medium *** *p* = 0.004; and 1.7 × 10^5^ RFU vs. 0.5 × 10^5^ RFU in SM^lowglu^ medium, **p = 0.0022; Figure 2B). Despite the increase in mROS levels in SM^lowglu^ medium in the presence of FLC, mROS levels were still lower when compared to mROS levels in cells grown in SM^nlglu^ in the presence of FLC (1.7 × 10^5^ RFU vs. 21.8 × 10^5^ RFU, **** *p* < 0.0001; Figure 2B).

One possible mechanism of lower mROS levels in cells grown in SM^lowglu^ medium is the altered expression of superoxide dismutases (SOD). SODs with the help of catalases convert reactive oxygen species to harmless water and free oxygen thereby lowering the oxidative stress in the cells [34]. Two SODs that regulate cellular and mitochondrial ROS are present in *C. neoformans*. *CNAG_04388* encodes for a SOD that is localized in the cytoplasm whereas *CNAG_01019* encodes for the SOD that is localized in mitochondria. The transcription of the mitochondrial SOD-encoding gene *CNAG_01019* was upregulated 11.3-fold (** *p* = 0.0063, Figure 2C), while the cytoplasmic SOD encoding gene *CNAG_04388* was up-regulated 5.4-fold (* *p* = 0.027, Figure 2C).

Another possible mechanism of lower mROS levels may be altered mitochondrial membrane potential (MMP). MMP regulates respiration rates and directly correlates with mROS [35,36]. MMP was measured by staining the cells with MitoTracker Deep Red that fluoresces in response to the MMP levels in mitochondria [37]. These data indicate a non-significant decrease in MMP in cells grown in SM^lowglu^ compared to those grown in SM^nlglu^ medium (1.2 × 10^6^ RFU vs. 1.9 × 10^6^ RFU, *p* = 0.0653; Figure 2D). However, in the presence of FLC, the MMP of cells grown in SM^nlglu^ medium significantly decreased when compared to the MMP of cells grown in SM^nlglu^ medium without FLC (0.2 × 10^6^ RFU vs. 1.9 × 10^6^ RFU; * *p* < 0.05, Figure 2D). In contrast, in cells grown in SM^lowglu^ medium in the presence or absence of FLC (Figure 2D) MMP remained comparable to the untreated cells. These data indicate that FLC induced a more profound mitochondrial dysfunction when cells were grown in SM^nlglu^ medium compared to SM^lowglu^ medium.

### 3.3. Low Glucose and FLC Exposure Alter Mitochondrial Morphology

**Figure 3:**
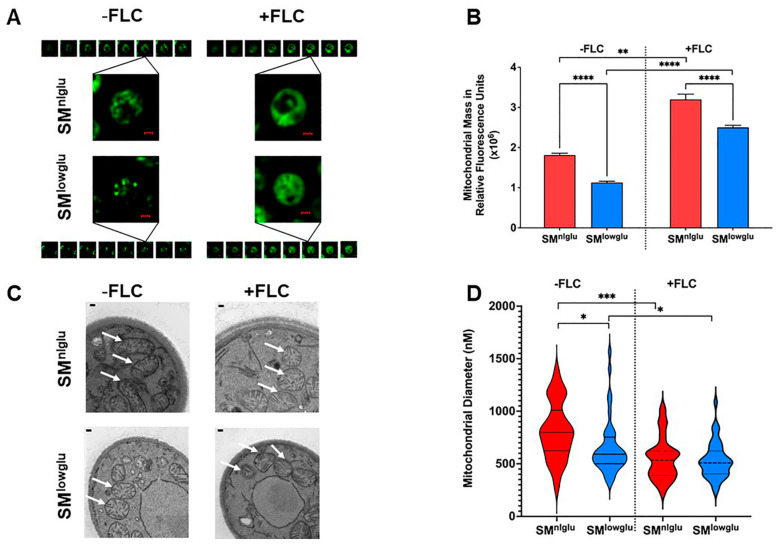
Effects of low glucose on mitochondrial structure. (**A**) Deconvolved fluorescent microscopy images show the difference in mitochondrial morphology in the presence (+FLC) and absence (−FLC) of 1 μg/mL FLC in KN99α cells in SM^nlglu^ and SM^lowglu^ media. Mitochondria were stained with MitoTracker Green FM. Each fluorescent field was imaged as a z-stack with 20 equal slices. Of the 20 slices, 8 slices for each condition are shown here. Scale bars in red = 1 µm. (**B**) Measurement of mitochondrial mass per 10^6^ KN99α cells grown in SM^nlglu^ (red bar) and SM^lowglu^ (blue bar) media in the presence (+FLC) or absence (−FLC) of 1 μg/mL of FLC. Mitochondria were stained with MitoTracker Green FM and fluorescence was measured. The assay was performed in biological triplicate. Error bars represent the standard deviation in the triplicate values. Student’s *t*-test with Welch’ correction was performed to determine the *p*-value (**** *p* < 0.0001, ** *p* = 0.0011). (**C**) TEM images of mitochondrial cross-sections of KN99α cells grown in SM^nlglu^ and SM^lowglu^ media in the presence (+FLC) or absence (−FLC) of 1 μg/mL of FLC showing different cross-sectional morphology of mitochondria. Scale bars in white = 100 nm. White arrows point to the mitochondrial structures as observed under TEM (**D**) Fiji was used to quantify the cross-sectional diameter of the mitochondria in SM^nlglu^ and SM^lowglu^ media in the presence (+FLC) or absence (−FLC) of 1 μg/mL of FLC. Forty mitochondria were measured from 30,000X zoom TEM images with a 500 nm scale bar. Student’s *t*-test with Welch’s correction was performed to analyze the *p*-values (* *p* < 0.05, *** *p* < 0.001).

Mitochondria make up 25% of the cell mass and they form complex networks that alter their morphology under stress by fusing and dividing [38]. Low glucose and FLC constitute specific forms of stress and their effect on mitochondrial morphology was explored. Mitochondria were stained with MitoTracker Green, which is a mitochondrial selective probe that covalently binds to mitochondrial proteins and accumulates in the mitochondrial matrix [39]. Effects of FLC on mitochondrial morphology were analyzed under normal (SM^nlglu^) and low glucose (SM^lowglu^ ) growth conditions by deconvolution fluorescence microscopy (Figure 3A).

These data highlight distinct differences in mitochondrial structures under the respective conditions (Figure 3A). In SM^nlglu^ medium, the mitochondrial structures resembled tubular networks whereas mitochondria were more fragmented in the cells grown in SM^lowglu^ medium. In contrast, in the presence of FLC, mitochondria were more dispersed throughout the cytoplasm and distinct mitochondrial structures were not visible (Figure 3A).

Next, total mitochondrial mass was measured by staining the mitochondria with MitoTracker Green dye and fluorescence intensity was measured using a spectrophotometer. These data demonstrated a lower total mitochondrial mass in cells grown in SM^lowglu^ when compared to the mitochondrial mass of cells grown in SM^nlglu^ (1.1 × 10^6^ RFU vs. 1.8 × 10^6^ RFU, **** *p* < 0.0001, Figure 3B). The addition of FLC increased the mitochondrial mass under both growth conditions, but it remained markedly reduced in cells grown in SM^lowglu^ medium compared to those grown in SM^nlglu^ medium (Figure 3B).

Next, a transmission electron microscopy (TEM) was used to examine the cross-section of the mitochondria in the cells grown in SM^nlglu^ and SM^lowglu^ media in the presence or absence of FLC. In the absence of FLC, the mitochondria of cells grown in SM^nlglu^ medium showed cylindrical cross-sections (Figure 3C). This was markedly different from the cross-sections of the mitochondria of cells grown in SM^lowglu^ medium, which exhibited a more circular cross-section (Figure 3C). In contrast, in the presence of FLC, mitochondria exhibited circular cross-sections in cells grown both in SM^nlglu^ and SM^lowglu^ media (Figure 3C).

Finally, Fiji was employed to quantify the diameters of the mitochondrial cross-sections. The mean mitochondrial diameter of the *C. neoformans* cells grown in SM^nlglu^ medium was significantly larger than the mean mitochondrial diameter of the cells grown in SM^lowglu^ medium (812.5 nm vs. 656.4 nm, * *p* < 0.05; Figure 3D). The mitochondrial size distribution was also more variable in cells grown in SM^nlglu^ medium when compared to the cells in SM^lowglu^ medium. In contrast, in FLC-treated cells, the mean mitochondrial diameter was smaller in cells grown under both conditions (Figure 3D). Specifically, in SM^nlglu^ medium with FLC, the mean mitochondrial diameter was 550.3 nm, *** *p* < 0.0001 (Figure 3D), which was ~1.5 fold lower than when grown without FLC. In SM^lowglu^ medium, the mitochondrial diameter was comparable at 533.3 nm, * *p* < 0.05, slightly less than (1.2 fold lower) than when grown in the absence of FLC. These findings are consistent with findings on fluorescence microscope images, which showed comparable dispersed mitochondrial morphology in cells grown in SM^nlglu^ and SM^lowglu^ media in the presence of sub-therapeutic FLC.

## 4. Discussion

The present study demonstrates that low glucose growth conditions greatly enhance FLC tolerance in *C. neoformans.* Glucose deprivation leads to enhanced FLC efflux through augmented efflux pump activity, marked changes of the mitochondrial network, and mitochondrial function, including lowered ROS accumulation and higher ATP production. In contrast to heteroresistance in *C. neoformans*, upregulation of Afr1 in the setting of glucose starvation is not associated with gene copy number variation. Although treatment with FLC compromises mitochondrial function, these data indicate that even in the presence of FLC treatment mitochondrial function is better preserved when *C. neoformans* cells are exposed to glucose starvation.

Low glucose remains one of the foremost stressors that *C. neoformans* encounters in the lung and CNS tissue [40,41,42,43,44], among a multitude of stresses during growth in the human host. For our investigations, a low glucose growth environment was achieved by substituting chemically defined medium with 0.05% glucose, which mimics the glucose concentration in vivo [21] and is substantially lower than the 2% glucose in standard fungal media. Our data demonstrate that under low glucose growth conditions, FLC tolerance is markedly increased (>32-fold) in laboratory strains as well as (4-fold) in low passage clinical *C. neoformans* strains. Increased FLC tolerance correlates with upregulation of *AFR1* and *AFR2* that encode for respective drug efflux pumps. Augmented efflux pump activity under low glucose conditions was confirmed by quantification of Nile Red efflux, which has been shown to correlate with efflux of FLC in *C. neoformans* [13], *Candida albicans* [45], and *S. cerevisiae* [46]. A previous study has demonstrated that Nile Red is the best substrate to assess the Afr1 pump in *C. neoformans* [13].

Afr1 has been identified as the most important membrane transporter in *C. neoformans* that exports FLC [13] and accordingly Δ*afr1* mutants exhibit a lower FLC MIC of 0.25 μg/mL when compared to the wild type parent MIC of 2 μg/mL [13]. Afr2 has also been shown to play a role in azole resistance, albeit less prominently [16]. The observed enhanced FLC tolerance in low glucose was lost in Δ*afr1* and Δ*afr1/*Δ*afr2* whereas Δ*afr2* mutant still exhibited increased FLC tolerance, which suggests that enhanced Afr1 efflux pump activity is responsible for the increased FLC tolerance under low glucose. Interestingly, Δ*mdr1* strain showed no increased FLC tolerance under low glucose conditions although *MDR1* transcription levels were not upregulated under low glucose conditions. It is conceivable that similar to reports from *Candida* sp. and *S. cerevisiae*, efflux pumps work in tandem, and basal level *MDR1* expression may be required in Afr1 mediated FLC tolerance under low glucose [47,48]. Alternatively, efflux efficacy could be altered through other mechanisms such as altered mRNA decay, protein translation, or even altered efflux pump localization [49].

*C. neoformans* strains are innately heteroresistant to FLC in vitro. It has been shown that heteroresistance occurs primarily through the formation of aneuploid cells [20,50,51]. The percentage of heteroresistant *C. neoformans* cells ranges from 0.01% to 25% in infected patients. A recent study examining the emergence of heteroresistance in FLC-treated patients [20] proposed that copy number variations of the Afr1 and Erg11 cause FLC tolerance. Unlike heteroresistance, FLC tolerance under glucose starvation is not limited to a subpopulation but instead the entire *C. neoformans* population exhibits higher tolerance for FLC. The 8.7-fold and 2.5-fold increase of *AFR1* and *AFR2* expression, respectively under low glucose is not associated with gene duplications, as reported in a high efflux clinical *C. neoformans* strain with a 2-fold expression increase resulting from disomy of Chr1 [51]. Possibly stress-induced upregulation of transcription factors is driving the augmented expression of genes encoding the efflux pumps. In support of this, we found that 6 of 20 transcription factors that have been previously implicated in mediating FLC resistance [13,52] are upregulated under low glucose (Table 3).

Afr1 and Afr2 are ABC transporters and enhanced pump efficacy requires an adequate supply of ATP, which is synthesized by mitochondria during respiration and fatty acid oxidation. Respiration produces 38 ATP molecules while fatty acid oxidation produces 129 ATP molecules. In *S. cerevisiae*, low glucose can trigger fatty acid oxidation [53]. Indeed, in our study, transcriptomic data under low glucose identified upregulation of several genes encoding key enzymes in the fatty acid oxidation pathway, which could explain the observed increased ATP levels under glucose starvation.

The majority of ATP is synthesized in the mitochondria. Loss of mitochondrial function has also been linked to FLC resistance in fungi. For instance, in *S. cerevisiae* and *C. glabrata*, loss of normal mitochondrial function led to upregulation of ABC transporter-encoding genes causing FLC resistance [54,55]. In *Aspergillus* sp. mutations of cofilin, a regulator of the actin cytoskeleton, trigger retrograde signaling and cause activation of the fatty acid oxidation pathway, resulting in increased cellular ATP and FLC tolerance [18].

Azoles cause oxidative damage in fungal cells by elevating mROS levels [56]. Cellular ROS levels are maintained by SODs [34], which catalyze the dismutation of superoxide anion-free O_2_- radical into molecular O_2_ and hydrogen peroxide thereby lowering the superoxide levels and preventing cellular damage [57]. Consistent with lower mROS levels, the cytosolic and mitochondrial SODs were upregulated under low glucose. As expected, mROS levels increased in response to FLC but were significantly less in cells grown under low glucose growth conditions. Compromised mitochondrial function can decrease MMP [58] and cause oxidative damage to proteins and mitochondrial DNA (mtDNA) [58]. In the presence of FLC, the MMP decreased in standard glucose growth conditions, whereas it remained unchanged in glucose-starved cells reflecting preserved mitochondrial function. Our findings are different from studies examining capsule induction in *C. neoformans* in low glucose growth conditions, which reported an increase in ROS and MMP. One difference is that those studies were done in 10% Sabouraud medium, which still contains 0.2% glucose, whereas our experiments were performed in synthetic defined medium supplemented with 0.05% glucose [59].

To maintain proper functionality, the mitochondrial network undergoes constant dynamic reorganization through fission and fusion, leading to a mitochondrial morphology that can range from reticular networks to highly fragmented mitochondria [38]. Mitochondrial fission causes fragmentation, which is mostly observed in resting cells [60,61,62]. We found that mitochondria displayed a reticular network in a normal glucose environment, which is consistent with previous findings [38]. Those studies described a distribution of distinct mitochondrial morphologies in *C. neoformans* populations exposed to H_2_O_2_ stress which we did not observe. However, although the entire fungal population exhibited a fragmented mitochondrial morphology under glucose starvation, a wide distribution of mitochondrial diameter was especially noticed in the untreated glucose starved cells. Fragmented mitochondria can fuse with neighboring mitochondria to maintain sufficient energy levels and prevent wide-scale damage [63,64], which leads to a decrease in mitochondrial mass [65]. Increased mitochondrial mass in normal glucose is explained by the higher oxidative stress [66,67]. It can also precede cell apoptosis [68] and although mitochondrial mass increased with FLC treatment, it was less in cells growing in low glucose, highlighting that this growth condition imposes lower stress on the mitochondria. In the presence of FLC, mitochondria were more dispersed and exhibited smaller median cross-sectional diameters than cells grown without drug. Studies in *C. glabrata* and *S. cerevisiae* have also shown that FLC compromises mitochondrial function [69]. Our data indicate FLC-induced mitochondrial damage is somewhat mitigated under low glucose conditions. To our knowledge, this is the first study, which has demonstrated that enhanced mitochondrial function may cause increased FLC resistance via enhanced efflux pump activity. Previous studies in *C. glabrata, C. albicans*, *S. cerevisiae* and *Aspergillus* sp. had shown the link between mitochondrial dysfunction and enhanced FLC resistance [17,18,70]. These studies showed enhanced expression of efflux pump encoding genes in cells with dysfunctional mitochondria [17,18,70].

**Figure 4:**
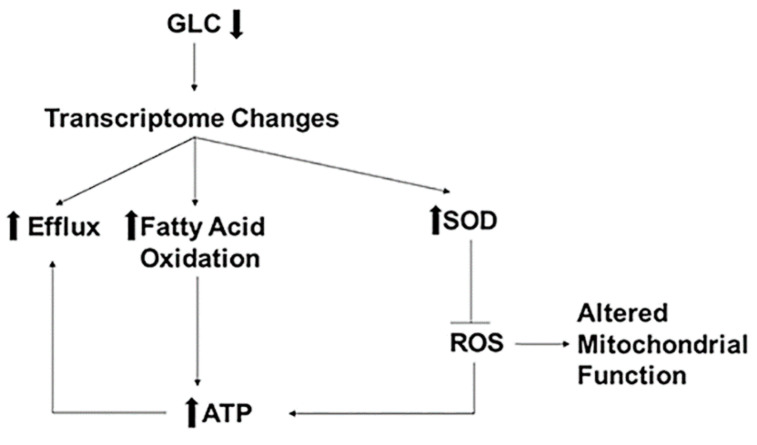
Low glucose induced mechanisms of FLC resistance. Low glucose (GLC) causes changes in the transcriptome, resulting in increased expression of genes encoding proteins for efflux, fatty acid oxidation, and superoxide dismutases (SOD). SODs inhibit ROS altering mitochondrial functions causing increased cellular ATP levels. Increased ATP levels can also be attributed to increased fatty acid oxidation. Increased ATP fuels the efflux pump causing increased FLC efflux resulting in increased FLC resistance.

Figure 4 and Table 4 summarize the findings of this study. Low glucose triggers transcriptome changes that cause increased expression of genes encoding drug efflux pumps, fatty acid oxidation, and SODs. Fatty acid oxidation under low glucose supplies the necessary ATP molecules to maintain enhanced efflux pump activity causing increased FLC tolerance. Additionally, increased gene expression of SODs may contribute to lowering the oxidative stress in the cells under low glucose. This aids the low glucose growing cells to combat FLC stress better than the normal glucose growing cells. Lastly, the findings also indicate that mechanisms for enhanced FLC tolerance in generationally aged cells may be more complex because replicative life span is also altered by low glucose growth conditions. Future experiments will have to differentiate between aging and carbon starvation mediated causes of enhanced FLC tolerance in generationally aged cells.

In summary, these investigations highlight how the simultaneous activation of stress pathways can contribute to emerging FLC tolerance. It appears that 0.05% glucose is a critical threshold governing FLC tolerance. FLC tolerance in the host environment may be underestimated if MICs are done in 2% glucose, which could contribute to the lack of correlation between MIC and outcome of CM. Finally, our data encourage efforts to further explore novel antifungal approaches that inhibit carbon starvation-induced stress.

## Figures and Tables

**Table 1:** Minimum inhibitory concentrations of different *C. neoformans* strains.

Strains	FLC (µg/mL)		
	2% GLC	0.5% GLC	0.05% GLC
KN99α	1	1	>64
H99	2	2	>64
RC2	2	2	>64
JEC21	2	2	>64
H99Δ*arf1*	0.25	NA	0.5
H99Δ*arf2*	2	NA	>64
H99Δ*mdr1*	2	NA	2
H99Δ*arf1*/Δ*arf2*	0.25	NA	0.25
I55	0.5	NA	2
I114	2	NA	8
J9	1	NA	2
J22	1	NA	4

NA—Not Applicable. Experiment not performed.

**Table 2:** Expression of genes encoding fatty acid oxidation proteins under low glucose compared to normal glucose.

Gene ID	Fold Change	*p*-Value	Description	Significant
CNAG_03080	0.70391347	1.77 × 10^−2^	fatty acid elongase, putative	YES
CNAG_01191	1.47264531	1.81 × 10^−2^	long-chain fatty acid transporter, putative	YES
CNAG_02099	1.68451118	7.76 × 10^−3^	fatty-acid synthase complex protein, putative	YES
CNAG_03019	1.92252186	2.61 × 10^−4^	long-chain-fatty-acid-CoA ligase, putative	YES
CNAG_00644	1.93712896	3.63 × 10^−6^	fatty acid desaturase, putative	YES
CNAG_04485	2.10846051	6.80 × 10^−5^	long-chain-fatty-acid-CoA-ligase, putative	YES
CNAG_00984	4.48453997	2.07 × 10^−5^	fatty acid beta-oxidation-related protein, putative	YES
CNAG_03666	6.42630743	2.31 × 10^−7^	acyl-CoA dehydrogenase, long-chain specific precursor, putative	YES
CNAG_04688	2.67314853	1.87 × 10^−4^	acyl-CoA dehydrogenase, putative	YES

**Table 3:** Expression of genes encoding the known transcription factors associated with FLC resistance under low glucose when compared to normal glucose.

Gene ID	Fold Change	*p*-Value	Description	Significant
CNAG_00896	10.4893255	3.49 × 10^−^^25^	transcription factor, putative	YES
CNAG_07724	3.42118669	4.18 × 10^−^^5^	ligand-regulated transcription factor, putative	YES
CNAG_04263	2.65694836	4.47 × 10^−^^3^	transcriptional activator, putative	YES
CNAG_00156	2.58714788	1.74 × 10^2^	conserved hypothetical protein	YES
CNAG_02696	2.87529851	3.56 × 10^−^^4^	exocyst protein, putative	YES
CNAG_01431	3.67805054	2.88 × 10^−^^6^	LIM-homeobox protein, putative	YES

**Table 4:** Impact of glucose on mitochondrial characterization in the presence or absence of fluconazole.

Mitochondrial Characterization	SM^nlglu^		SM^lowglu^	
	(−) FLC	(+) FLC	(−) FLC	(+) FLC
ATP levels	↓	↓	↑↑	↑
mROS	↑	↑↑	↓↓	↓
SOD	↓	nt	↑↑	nt
MMP	↑↑	↑	↓	↑
Mitochondrial morphology	tubular	dispersed	fragmented	dispersed
Mitochondrial mass	↑	↑↑	↓↓	↓
Mitochondrial diameter	↑	↓	↓	↓↓

SM: synthetic media; (^nlglu^): normal (2%) glucose; (^lowglu^): low (0.05%) glucose; FLC: fluconazole; ATP: adenosine triphosphate; mROS: mitochondrial reactive oxygen species; SOD: superoxide dismutase; MMP: mitochondrial membrane potential; nt: not tested. Up arrows signify increased levels. Down arrows signify decreased levels. Double up arrows signify that the levels further increased compared to no drug conditions. Double down arrows signify that the levels further decreased compared to no drug conditions.

## Data Availability

All data required to understand this article are presented in the study or the Supplementary Materials. Any raw data further requested will be provided from the corresponding authors.

## Supplementary Materials

The following are available online at https://www.mdpi.com/article/10.3390/jof7060489/s1: Figure S1—Gene Duplication; Table S1—List of Strains; Table S2—Oligonucleotides Used; Table S3—Ergosterol Gene Expression.
