# Supplementary material for: Low Glucose Mediated Fluconazole Tolerance in Cryptococcus neoformans
Source: J Fungi (Basel). 2021 Jun 18;7(6):489. doi: 10.3390/jof7060489 (PMC8233753; doi:10.3390/jof7060489)
Supplement: Supplementary file 1 [file jof-07-00489-s001.zip › Table S1_List of Strains.pdf]

Table S1. Strains used in the study.

C. neoformans strains	Serotype	Source or Reference
H99	A	WT
KN99α	A	WT
RC2	D	ATCC 24067 variant
JEC21	D	WT
H99Δ*afr1*	A	(1)
H99Δ*afr2*	A	(1)
H99Δ*mdr1*	A	(1)
H99Δ*afr1*/Δ*afr2*	A	(1)
I55	A	(2)
I114	D	(2)
J9	D	(2)
J22	D	(2)

1. Chang M, Sionov E, Khanal Lamichhane A, Kwon-Chung KJ, Chang YC. 2018. Roles of Three Cryptococcus neoformans and Cryptococcus gattii Efflux Pump-Coding Genes in Response to Drug Treatment. Antimicrob Agents Chemother 62.
2. Bouklas T, Diago-Navarro E, Wang X, Fenster M, Fries BC. 2015. Characterization of the virulence of Cryptococcus neoformans strains in an insect model. Virulence 6:809-13.
