# Supplementary material for: Low Glucose Mediated Fluconazole Tolerance in Cryptococcus neoformans
Source: J Fungi (Basel). 2021 Jun 18;7(6):489. doi: 10.3390/jof7060489 (PMC8233753; doi:10.3390/jof7060489)
Supplement: Supplementary file 1 [file jof-07-00489-s001.zip › Table S2_Oligonucleotides_Used.pdf]

Table S2. Oligonucleotides Used

Gene Name	Forward (5′-3′)	Reverse (5′-3′)
ACT1	CCCACACTGTCCCCATTTAC	AACCACGCTCCATGAGAATC
SIR2	ATGAAGAACGCCATTTCCAC	CTAGGCGGTTCATCAGCTTC
CNAG_01019	AACACCAACGGCTGTACCTC	TTCGACCAATGATGGAGTGA
CNAG_04388	TTCCCACCTCTGGTGTCTTC	TAGAAAGCGTGCTCCCAGAT
AFR1	CTTTCCGAGCTGGTGAACTC	CACCTTCGATCACACCAATG
AFR2	GGTTCCGACTACATGGCTGT	GAGTTCACCAGCTCGGAAAG
MDR1	CTCTTGATCACATCGCGAAA	ACCGACAATCTTGCTCTGCT
ERG11	CTGGCCAGCCATAAGAAGAG	GTGAAAGCGACGTGAGTTGA
