# Supplementary material for: Low Glucose Mediated Fluconazole Tolerance in Cryptococcus neoformans
Source: J Fungi (Basel). 2021 Jun 18;7(6):489. doi: 10.3390/jof7060489 (PMC8233753; doi:10.3390/jof7060489)
Supplement: Supplementary file 1 [file jof-07-00489-s001.zip › Table S3_Ergosterol_Gene_Expression.pdf]

Table S3: Expression of genes encoding ergosterol biosynthesis enzymes under low glucose compared to normal glucose

Gene ID	Description	logFC	P-Value	Significant
CNAG_03009	ergosterol biosynthesis-related protein, putative	-0.86773	2.03×10^{-3}	YES
CNAG_02830	delta24(24-1) sterol reductase, putative	-0.83555	2.89×10^{-5}	YES
CNAG_04605	C-3 sterol dehydrogenase (C-4 sterol decarboxylase), putative	-0.80161	4.77×10^{-5}	YES
CNAG_00519	C-5 sterol desaturase, putative	-2.00565	9.68×10^{-19}	YES
CNAG_00854	C-8 sterol isomerase, putative	-1.33191	1.33×10^{-8}	YES
CNAG_01737	C-4 methyl sterol oxidase, putative	-1.06812	1.98×10^{-4}	YES
CNAG_00040	sterol 14-demethylase, putative	-1.00762	3.04×10^{-9}	YES
CNAG_03819	sterol 24-C-methyltransferase, putative	-1.06486	7.61×10^{-7}	YES
CNAG_02896	hydroxymethylglutaryl-CoA synthase, putative	-0.78075	2.41×10^{-6}	YES
CNAG_06534	hydroxymethylglutaryl-CoA reductase (NADPH), putative	1.07213	4.16×10^{-5}	YES
