# Supplementary figures and images for: Low Glucose Mediated Fluconazole Tolerance in Cryptococcus neoformans
Source: J Fungi (Basel). 2021 Jun 18;7(6):489. doi: 10.3390/jof7060489 (PMC8233753; doi:10.3390/jof7060489)

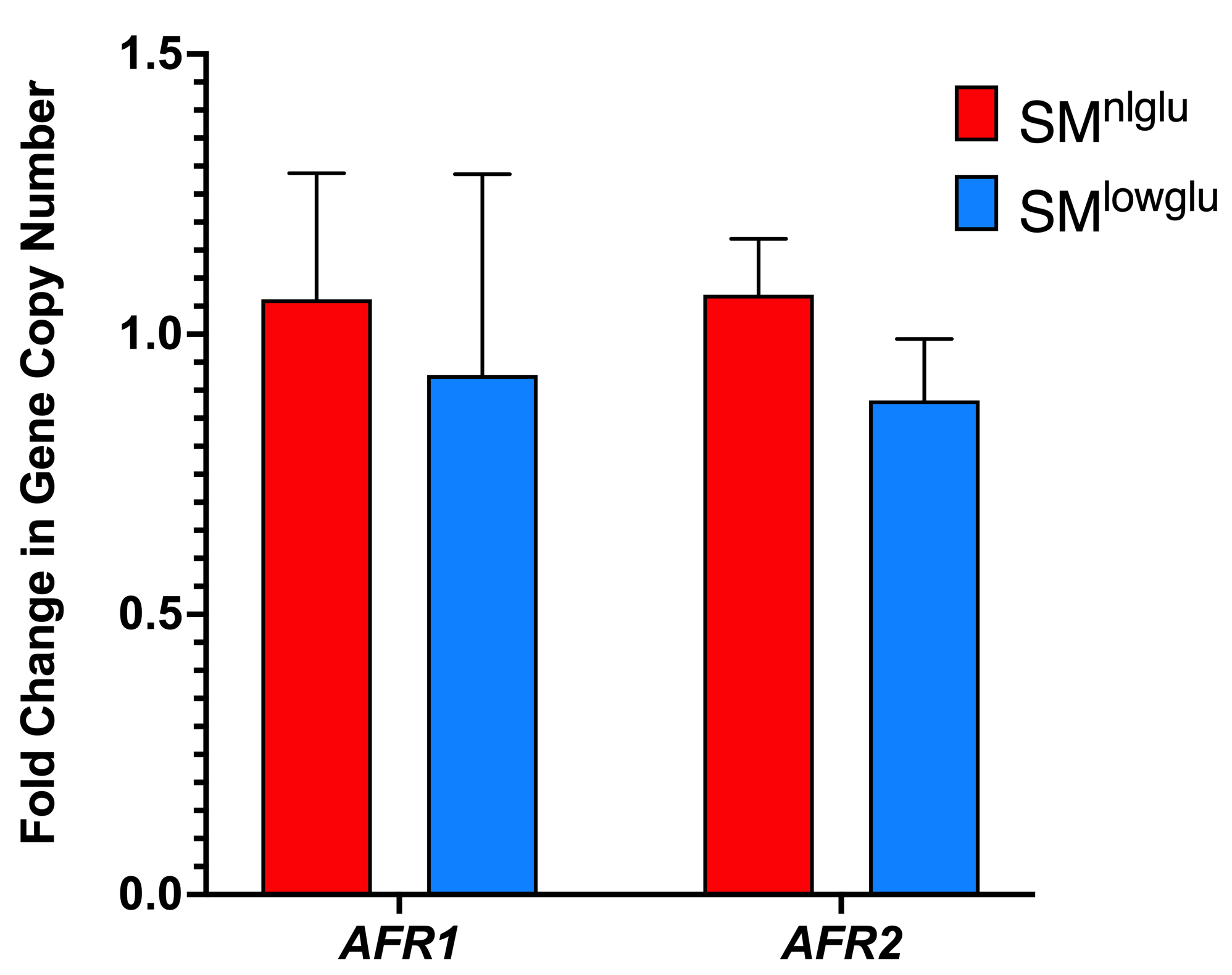

Supplement: Supplementary file 1 [file jof-07-00489-s001.zip › Fig S1_Gene_Duplication.tiff]
